# miR-339-5p inhibits cell migration and invasion *in vitro* and may be associated with the tumor-node-metastasis staging and lymph node metastasis of non-small cell lung cancer

**DOI:** 10.3892/ol.2014.2165

**Published:** 2014-05-22

**Authors:** YUN LI, WEIGUO ZHAO, PENGTAO BAO, CHUNSUN LI, XIU QING MA, YANQING LI, LIANG AN CHEN

**Affiliations:** 1 Department of Respiratory Medicine, Chinese PLA General Hospital and Chinese PLA Medical School, Beijing 100853, P.R. China; 2 Department of Respiratory Medicine, The 309th Hospital of Chinese People’s Liberation Army, Beijing 100091, P.R. China

**Keywords:** microRNAs, tumor-node-metastasis staging, lymph node metastasis, lung carcinoma

## Abstract

The aim of the present study was to investigate the potential role of microRNA (miRNA or miR) in invasion and metastasis of non-small cell lung cancer (NSCLC). miRNA-microarray analysis was used to detect the differentially expressed miRNAs between various metastatic levels of NSCLC cells. The microarray results were verified by quantitative polymerase chain reaction. The most clearly altered miRNA, miR-339-5p, was transfected into NSCLC cells and cell migration and invasion were investigated. The expression of miR-339-5p was 3.4662-fold higher in the lower metastatic NSCLC cells. miR-339-5p significantly decreased tumor-cell migration and the invasion capacity *in vitro*. In conclusion, miR-339-5p is important in NSCLC invasion and metastasis, indicating that miR-339-5p could be further evaluated as a biomarker for predicting the survival time of patients with NSCLC.

## Introduction

Lung cancer is the most prevalent cause of cancer-related mortality worldwide, and non-small cell lung cancer (NSCLC) is responsible for ~80% of all lung cancer cases (1). microRNAs (miRNAs or miRs) are a class of endogenous, non-coding, single-stranded RNAs with a length of 20–25 nucleotides. miRNA genes are transcribed in the nucleus to primary transcripts (2). Following digestion by Drosha, precursor miRNA is formed and transported to the cytoplasm, where it is digested by Dicer to produce mature miRNA (3,4). miRNAs are believed to regulate other genes by hybridizing to complementary sequences in the 3′ untranslated region (3′UTR) of target mRNA, resulting in mRNA degradation or translational inhibition (5).

Previously, numerous studies have demonstrated that miRNAs play a significant role in the development and progression of NSCLC. A study by Yanaihara *et al* (6) reported that patients with lung cancer with a high expression of miR-155 or low expression of miR-let-7a-2 exhibited a worse prognosis. The miRNA-200 family members have been shown to affect E-cadherin expression and epithelial-to-mesenchymal transition, which is an essential early step in tumor metastasis (7). A study by Ceppi *et al* (8) reported that the loss of miR-200c expression induced an aggressive, invasive and chemoresistant phenotype in NSCLC. Understanding the specific roles of miRNAs in NSCLC progression could aid in identifying predictive markers and devising novel therapeutic strategies for patients.

In the present study, the potential roles of miRNA in invasion and metastasis of NSCLC were investigated. First, miRNA microarray analysis was performed to identify the various expressions of miR-339-5p in different NSCLC cells. Subsequently, NSCLC cell migration and invasion assays were performed *in vitro*. Finally, the tissue samples were used to validate these results. Quantitative polymerase chain reaction (qPCR) analysis was performed in three independent experiments, each using two independent samples. miRNA expression data are presented as fold difference relative to U6 based on the following equation: RQ = 2^−ΔΔCt^.

## Materials and methods

### Cell culture

Paired high-metastatic human pulmonary giant cell carcinoma cells, 95D, and low-metastatic human pulmonary giant cell carcinoma cells, 95C, were provided by the laboratory of the Department of Respiratory Diseases [Chinese People’s Liberation Army (PLA) General Hospital, Beijing, China] and grown in RPMI-1640 medium (Gibco, Carlsbad, CA, USA) with 10% fetal bovine serum (FBS; Gibco) at 37°C, in a humidified atmosphere of 95% air and 5% CO_2_. This study was approved by the ethics committee of The 309th Hospital of Chinese People’s Liberation Army (Beijing, China)

### NSCLC tissue specimens

A total of 60 surgical NSCLC tissue specimens and paired adjacent normal lung tissues (NAT) were obtained from the Chinese PLA General Hospital and Chinese PLA 309th Hospital (Beijing, China). Patients who had received any chemotherapy or radiation therapy prior to surgery or had rheumatic disease, acute infection, human immunodeficiency virus or other types of cancer were excluded from the present study. Clinical stage was determined according to the American Joint Commission on Cancer and Union for International Cancer Control 2007 tumor-node-metastasis (TNM) staging criteria (9). RNA was extracted and qPCR was performed.

### Isolation of total RNA

RNA of the NSCLC cells and fresh tissue samples were extracted using the mirVana RNA isolation kit (Am1560; Ambion, Austin, TX, USA) according to the manufacturer’s instructions. RNA quality and quantity was determined by spectrophotometry (ND-1000; NanoDrop Technologies, Wilmington, DE, USA).

### miRNA microarray

The total RNA was phosphorylated and dimethyl sulfoxide was added to dephosphorylate the RNA. Subsequently, the mixture was assembled and the reaction was labeled and incubated for 2 h at 16°C. The sample was then dried with a vacuum concentrator for 1–2 h at 45–55°C. The hybridization mixture was then assembled according to the manufacturer’s instructions. It was subsequently delivered to the chip, which was covered with Agilent human miRNA array V12.0 (Agilent Technologies, Inc., Santa Clara, CA, USA), prior to drying for 20 h at 55°C and 20 × g. Finally, the miRNA microarray was washed and scanned by an Agilent microarray scanner (Agilent Technologies, Inc.).

### qPCR

RNA samples of lung cancer cells or tissue samples were subjected to reverse transcription reactions using the TaqMan microRNA reverse transcription kit (4366596; Ambion). Subsequently the cDNA was amplified by qPCR using the TaqMan Assay (miRNA339-5p, 4427975; and U6, 439547; Ambion) with the TaqMan Universal Master Mix (4369016; Ambion) in three independent experiments, each using three independent samples. U6 small nuclear RNA was used as an internal control. miRNA expression data are presented as the fold difference relative to U6 based on the following equation: RQ = 2^−ΔΔCt^, where RQ is the relative quantity.

### Transient miRNA transfection

95C and 95D cells (1×10^6^) were seeded and grown overnight in six-well plates. The following day, the cells were transfected with either the miR-339-5p mimic, 2′-O-methylated single-stranded miR-339-5p antisense oligonucleotides (ASO) or the control oligonucleotides (GenePharma Co., Ltd., Shanghai, China) using Lipofectamine 2000 (Invitrogen Life Technologies, Carlsbad, CA, USA), according to the manufacturer’s instructions. The miRNA mimics are small double-stranded RNA oligonucleotides, with the sequence 5′-UCCCUGUCCUCCAGGAGCUCACGUGAGCUCCUGGAGGACAGG GAUU-3′. The ASO sequence was 5′-CGUGAGCUCCUGGAGGACAGGGA-3′. The negative control RNA was used to eliminate the potential non-sequence-specific effects, and the sequences were non-homologous to any human genome sequences. Those sequences were 5′-UUCUCCGAACGUGUCACGUTT-3′ (sense), 5′-ACGUGACACGUUCGUAGAATT-3′ (antisense; a negative control for the miRNA mimic) and 5′-CAGUACUUUUGUGUAGUACAA-3′ (a negative control for the mRNA antisense transfection).

### Cell migration and invasion assay

A Transwell insert (24-well insert, pore size 8 μm; Corning, Inc., Corning, NY, USA) was used to investigate the effect of miR-339-5p on the migration and invasion of the 95C and 95D cells *in vitro*.

For the cell migration assay, 4×10^4^ cells were resuspended in serum-free RPMI-1640 and placed in the top portion of the chamber. The lower chamber was filled with 10% FBS as the chemoattractant and incubated at 37°C in 5% CO_2_ for 24 h. Subsequently, the cells on the upper surface of the membrane were removed using cotton buds with phosphate-buffered saline, and the cells on the lower surface of the insert were fixed in 75% methanol and stained with 0.1% crystal violet. The images of five random fields of each insert were captured under a light microscope at a magnification of ×200 (Nikon Corporation, Tokyo, Japan). The cells in the images were counted, and the data were summarized as the means ± SD and presented as a percentage of the controls. Assays were conducted in duplicate in three independent experiments.

For the invasion assay, the inserts were previously covered with 100 μl of the mixture, which contained pre-cooled serum-free RPMI-1640 and Matrigel (1:10; BD Biosciences, San Diego, CA, USA), and were allowed to solidify at 37°C in 5% CO_2_ for 3 h. Following this, 5×10^4^ cells were resuspended in serum-free RPMI-1640 and placed in the top portion of the chamber and then the remainder of the invasion assay followed the protocol for the cell migration assay.

### Statistical analysis

All statistical analyses were performed using SPSS 13.0. The paired-samples t-test was used to analyze significant differences in has-miR-339-5p expression between NSCLC and NAT tissues. The χ^2^ test was used to determine the correlation between has-miR-33p-5p expression and clinicopathological variables. The two-sided Fisher’s exact test was used to determine the association between has-miR-339-5p expression and clinicopathological variables when the number of tumors analyzed was less than five. The Mann-Whitney U test was used for clinical-stage ranked data analysis. Spearman’s correlation analysis was used to determine the correlation between miR-339-5p expression and clinical stage and lymph node metastasis status. Other results were analyzed using the independent samples t-test. Results were considered to indicate a statistically significant difference at values of P<0.05.

### Prediction of miR339-5p target genes

Three miRNA databases (http://www.microrna.org/microrna/home.do; http://pictar.mdc-berlin.de; and http://www.targetscan.org) were searched for prediction of miR-339-5p target genes.

## Results

### Differential miRNAs between 95C and 95D cells by microarray analysis

Agilent human miRNA array V12.0, which contained 855 probes based on Sanger miRBase release 13.0, was used to scan miRNAs that were differentially expressed between 95C and 95D cells. The cells were paired pulmonary giant cells with low or high metastatic capacities, respectively. In total, 44 miRNAs exhibited significantly differential expression, among which miR-339-5p was focused on as it is one of the most evidently altered miRNAS and has previously been reported to be associated with the metastasis of breast cancer (10) (Table I).

### qPCR verification of miR-339-5p expression between 95C and 95D cells

**Figure 1:**
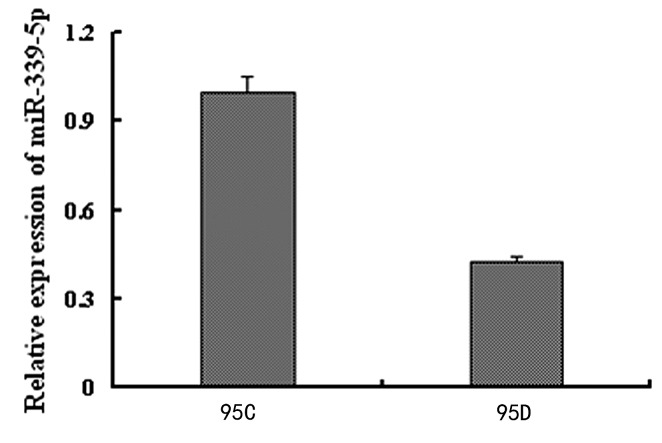
95C and 95D cells are the sublines of human lung giant-cell carcinoma maternal cells (PLA-801). The paired cells have the same genetic background and varied metastatic capacity. 95D cells had the higher metastatic capacity. Quantitative polymerase chain reaction validated that miR-339-5p expression of the 95C cells was 34,662-fold higher compared with that of 95D cells. miR, microRNA.

qPCR was further used to validate that the miR-339-5p expression of the 95C cells was significantly higher, by 3.4662 fold, compared with that of 95D cells (Fig 1).

### Effects of miR-339-5p on cell migration and invasion

**Figure 2:**
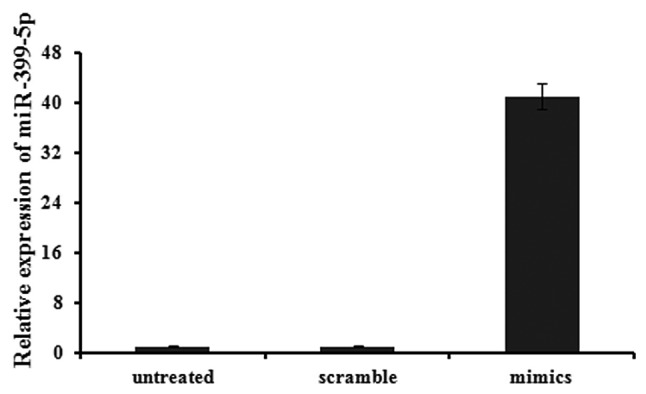
miR-339-5p mimics and a negative control were transfected into the 95D cell line, respectively. Quantitative polymerase chain reaction confirmed that the miR-339-5p expression of the 95D cell line increased, by 41.07 fold, 24 h after transfection of miR-339-5p mimics. miR, microRNA.

**Figure 3:**
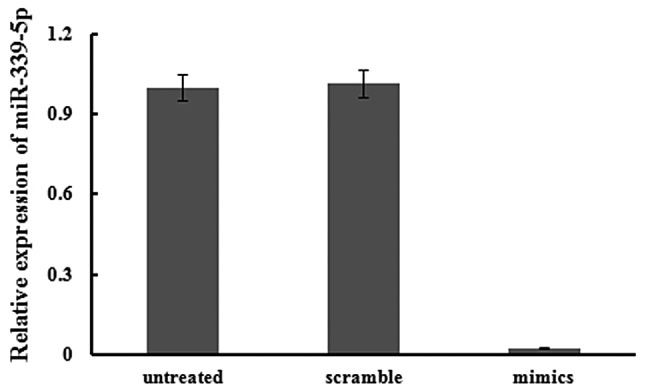
miR-339-5p mimics and a negative control were transfected into the 95C cell line, respectively. Quantitative polymerase chain reaction confirmed that the miR-339-5p expression of the 95C cell line decreased, by 38.73-fold, 24 h after transfection of miR-339-5p mimics. miR, microRNA.

qPCR was used to confirm that transfection was successful. Expression in transfected cells was normalized to that of untreated cells, and U6 expression was used as an internal standard. The expression of has-miR-339-5p was clearly increased, by 41.07 fold, by transfection of miR-339-5p mimics into 95D cells after 24 h (Fig. 2). Simultaneously, the expression of has-miR-339-5p was markedly decreased, by 38.73 fold, by transfection of the ASO of miR-339-5p into 95C cells after 24 h (Fig. 3).

### Effects of miR-339-5p on NSCLC cell migration and invasion

**Figure 4:**
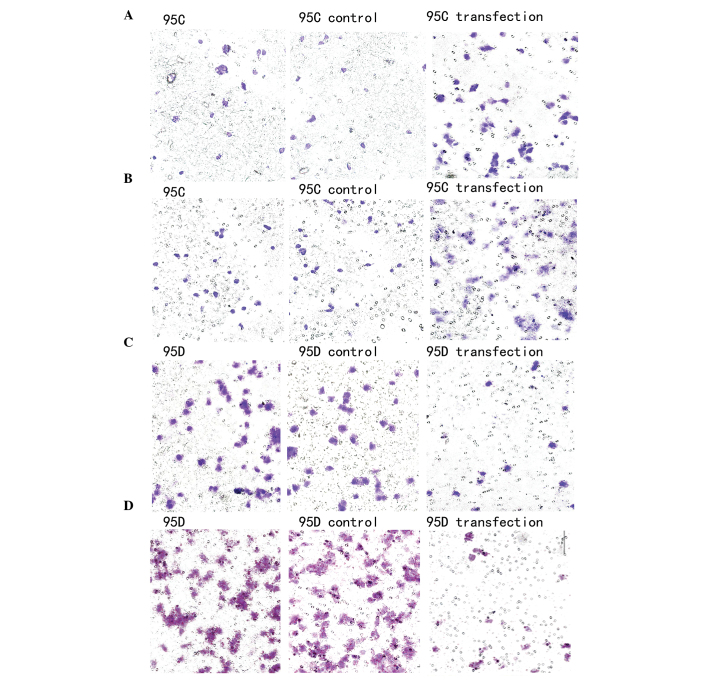
Migration and invasion assays of the 95C and 95D cells. (A) In the migration assay, there were no differences between untreated 95C cells and the control cells which were transfected with the scramble oligonucleotide (35±3 and 37±4 cells, P=0.814). However, the number of migrating cells that were transfected with the antisense oligonucleotides (ASO) was significantly increased (56±5 cells, P<0.01). (B) In the invasion assay, there were no differences between untreated 95C cells and the control cells which were transfected with the scramble oligonucleotide (25±2 and 22±4 cells, P=0.768). However, the number of invading cells that were transfected with the ASO was significantly increased (50±3 cells, P<0.01). (C) In the migration assay, there were no differences between untreated 95D cells and the control cells which were transfected with the scramble oligonucleotide (55±3 and 51±4 cells, P=0.814). However, the number of migrating cells that were transfected with the mimics was significantly decreased (10±5 cells, P<0.01). (D) In the invasion assay, there were no differences between untreated 95D cells and the control cells which were transfected with the scramble oligonucleotide (70±2 and 67±4 cells, P=0.768). However, the number of invading cells that were transfected with the mimics was significantly decreased (12±3 cells, P<0.01).

A loss-of-function approach was adopted for the analysis of the effects of miR-339-5p on NSCLC cell migration and invasion. The expression of has-miR-339-5p was decreased by transfection of 2′-O-methylated single-stranded miR-339-5p ASO into 95C cells, which was validated by qPCR. There were no variations between untreated cells and cells transfected with the scramble oligonucleotide (P=0.814). However, the number of migrating cells that were transfected with the ASO was significantly increased (P<0.01) (Fig 4A). For the invasion analysis, there was no difference between untreated cells and cells transfected with the scramble oligonucleotide. However, the number of invading cells that were transfected with the ASO was significantly increased (P<0.01) (Fig 4B).

A gain-of-function approach was then adopted. The expression of has-miR-339-5p was increased by transfection of the miR-339-5p mimics into 95D cells and validated by qPCR. There were no variations between untreated cells and cells transfected with the scramble oligonucleotide (P=0.814). However, the number of migrating cells that were transfected with the mimics was significantly increased (P<0.01) (Fig 4C). For the invasion analysis, there was no variation between untreated cells and cells transfected with the scramble oligonucleotide (P=0.768). However, the number of invading cells that were transfected with the mimics was significantly increased (P<0.01) (Fig 4D).

### Decreased expression of miR-339-5p in NSCLC cancer tissues

**Figure 5:**
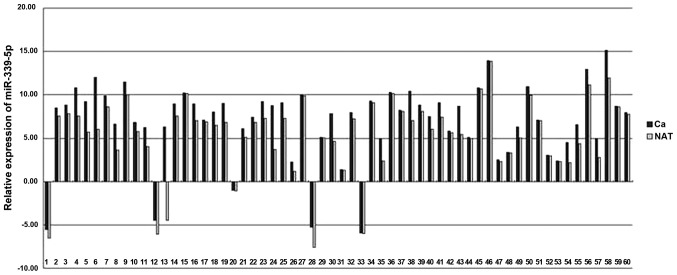
Decrease in relative expression of miR-339-5p in 60 non-small-cell lung cancer (NSCLC; Ca) tissues in comparison with corresponding normal adjacent tumor tissues (NATs). The data are representative of three independent experiments and the relative expression values were calculated using the equation: RQ = 2^−ΔΔCt^, where RQ is the relative quantity. The mean expression level of miR-339-5p in NSCLCs was decreased by ~1.9 fold compared with that in NATs (minimum, 33.78 fold; and maximum, 1.03 fold). miR, microRNA.

In the patients with NSCLC, miR-339-5p expression was decreased in cancer tissues in comparison with matched LACs (Fig 5). The mean expression levels of miR-339-5p in NSCLCs was decreased by ~1.9 fold compared with NATs (minimum, 33.78; and maximum, 1.03).

### Association of has-miR-339-5p relative quantitative expression (cancer tissue expression/normal tissue expression) in NSCLCs and NATs with clinicopathological features of NSCLCs

To determine the effects of has-miR-339-5p expression on tumor progression and metastasis, the patients with lung cancer were divided into two groups based on the mean level of the ratio of miR-339-5p relative expression (carcinoma/NAT) in 60 NSCLCs (mean =0.5249). The two groups were defined as high-relative and low-relative expression (≥0.5249 or <0.5249). The associations between miR-339-5p relative expression and clinicopathological variables for lung cancer are shown in Table II. Associations between miR-339-5p expression and lymph node metastasis were observed to be statistically significant (P<0.001, two-sided Fisher’s exact test). Changes in expression of miR-339-5p were also statistically significantly associated with clinical stages (P<0.001, Mann-Whitney test). No correlation was observed between miR-339-5p expression and gender, age and pathological type (data not shown).

In order to improve the characterization of the association between miR-339-5p expression, TNM stage and lymph node metastasis, the data was further analyzed using Spearman’s correlation test. The results showed a negative correlation between has-miR-339-5p relative quantitative expression and TNM stage (r=−0.927, P<0.001) and lymph node metastasis (r=−0.828, P<0.001).

### Bioinformatic analyses

Bioinformatic analyses found that B-cell lymphoma 6 protein (BCL6) and valosin-containing protein (VCP) gene may be potential miR-339-5p targets.

## Discussion

Metastasis is a common event in cancer pathology and represents the primary cause of cancer-related mortality. The steps required for metastasis involve significant changes in gene expression. A number of studies investigating miRNA regulation of the aforementioned steps have identified several miRNAs that may promote or inhibit the metastatic potential. It has been reported that miRNA-10b, miR-21, miR-373, miR-378 and miR-17-92 can promote breast cancer metastasis (11–15), while miR-335, miR-206 and let-7 family can inhibit the metastasis of breast cancer (16,17). Although there have been numerous studies on the miRNAs associated with lung cancer metastasis (6,8), but the molecular mechanism is not clear.

miRNA microarray analysis is a high-throughput rapid analysis of the miRNA expression profiling method. 95C and 95D cells were the sublines of human lung giant cell carcinoma maternal cells (PLA-801) that were isolated by the Department of Pathology, of the General Hospital of Chinese PLA Hospital (Beijing, China). The paired cells have the same genetic background and varied metastatic capacity. In order to study the miRNAs associated with the NSCLC metastasis, the miRNA microarray was first applied to find 44 miRNAs whose expression were different in the 95C and 95D cells. Compared with 95C cells, 29 miRNA expression levels of 95D increased and 15 miRNAs were downregulated, which included miRNA-339-5p. The expression of miRNA-339-5p in 95C cells was eight-fold higher compared with that in the 95D cells. Subsequently, qPCR detection of the miRNA-339-5p expression levels of 95C and 95D cells was performed, and the results of which were consistent with the microarray results. Transfection, migration and invasion assays were then used to confirm that miRNA-339-5p could inhibit the NSCLC cell migration and invasion ability.

Finally, qPCR confirmed that the expression of has-miR-339-5p was decreased significantly in the majority of NSCLCs. A negative correlation was evaluated between has-miR-339-5p relative quantitative expression and TNM stage and lymph node metastasis. These data indicate that has-miR-339-5p could inhibit NSCLC metastasis.

miRNAs regulate gene expression by binding to sequences in the 3′UTR of an expressed mRNA, resulting in either modulation of translation efficiency or degradation of the mRNA. In order to understand the mechanism of miRNA inhibiting metastasis, the interaction between miRNA and target genes must be known. miRecords is one of the most commonly used mRNA target gene predicting sites (18). The site integrates information from numerous target gene predictable sites. The genes that were predicted by the majority of sites from miRecords were chosen as the target genes in the present study. The sites must include TargetScan, PicTar and miRanda, which are the three most commonly used bioinformatic programmes to predict miRNA target genes (19–21). According to the predicted results from the webserver databases, BCL6 and VCP were the most likely target genes predicted. BCL6 is a proto-oncogene located on chromosome 3q27 that encodes a transcriptional repressor that was originally characterized as a regulator of B-lymphocyte development and growth and has been indicated in the pathogenesis of B-cell lymphoma (22,23). Numerous studies have revealed that BCL6 is associated with cancer metastasis. A study by Pinto *et al* (24) found that BCL6 could significantly increase the expression of three metastasis-related genes [chemokine (CXC motif) receptor 4, fms-related tyrosine kinase 1 and integrin β3] in breast cancer cell lines. A study by Wu *et al* (10) revealed that miR-339-5p could inhibit the expression of BCL-6 mRNA, which is associated with suppression of the migration and invasion of breast cancer cells. Further studies are required to find the association between BCL6 and lung cancer metastasis. VCP (also known as p97) is a member of the AAA ATPase family. VCP has a pivotal role in the ubiquitin-degradation of misfolded proteins and also exhibits an anti-apoptotic function and metastasis via the activation of the nuclear factor-κB (NF-κB) signaling pathway. Yamamoto *et al* (25) found that VCP (p97)expression is associated with the progression and prognosis of patients with NSCLC. VCP is also active in the ubiquitin/proteasome-degradation pathway, which is involved in proliferation and anti-apoptosis in human cancer cells. Studies have shown that the expression levels of VCP correlate with the prognosis and recurrence of specific human cancers, including hepatocellular, gastric and colorectal carcinomas. In these studies, VCP expression was found to correlate with the prognosis of differentiated thyroid carcinoma (26–28). It is speculated that miR-339-5p inhibits the VCP expression to inhibit the metastasis of lung cancer, but the exact mechanism is unclear.

In conclusion, the results of the present study identified that miR-339-5p was significantly downregulated in the primary tissues of patients with NSCLC compared with adjacent normal tissues. The strong correlation between miR-339-5p expression and the clinical stage indicates that miR-339-5p may be a novel biomarker involved in lung cancer metastasis, but further studies are required to reveal the exact mechanism.

## Figures and Tables

**Table I:** Fold changes of the miRNA expression between 95C and 95D cells.

Systematic name	Fold-change (95C vs. 95D)	Regulation (95C vs. 95D)
hsa-miR-146b-3p	5.7694	Up
hsa-miR-513a-3p	6.2644	Up
hsa-miR-155	2.1363	Up
hsa-miR-338-5p	2.0583	Up
hsa-miR-588	8.1228	Up
hsa-miR-924	4.3476	Down
hsa-miR-494	2.1707	Down
hsa-miR-339-5p	8.0135	Up
hsa-miR-150^*^	5.3655	Down
hsa-miR-1226^*^	2.3421	Down
hsa-miR-493^*^	3.7905	Up
hsa-let-7g	2.2086	Up
hsa-miR-30e^*^	2.3531	Up
hsa-miR-106b^*^	2.3968	Down
hsa-miR-1299	5.1606	Up
hsa-miR-1915	2.1175	Down
hsa-miR-30a^*^	2.2098	Up
hsa-let-7b	2.0122	Up
hsa-miR-28-5p	2.5076	Up
hsa-miR-1287	8.3396	Down
hsa-miR-26b	2.0724	Up
hsa-miR-1290	3.2220	Up
hsa-miR-892a	6.3257	Up
hsa-miR-7	2.2993	Up
hsa-miR-1826	10.5247	Down
hsa-let-7f	2.0995	Up
hsa-miR-198	16.6237	Down
hsa-miR-658	23.5899	Down
hsa-miR-1246	2.9612	Up
hsa-miR-299-5p	2.0088	Up
hsa-miR-29b-1^*^	2.1965	Up
hsa-miR-760	7.0691	Up
hsa-miR-923	2.2522	Down
hsa-miR-630	4.5866	Down
hsa-miR-501-5p	2.0365	Up
hsa-miR-324-5p	2.0318	Down
hsa-miR-196a	2.2921	Up
hsa-miR-196b	2.4324	Up
hsa-miR-513b	3.3342	Down
hsa-miR-129^*^	2.3897	Up
hsa-miR-92a-2^*^	20.1876	Down
hsa-miR-625	2.2465	Up
hsa-miR-624	4.8870	Up
hsa-miR-1268	6.8535	Up

Agilent Human miRNA array V12.0, which contained 855 probes based on Sanger miRBase release 13.0, was used to scan miRNAs that were differentially expressed between 95C and 95D cells. These were the 44 miRNAs which exhibited significantly differential expression. miRNA or miR, microRNA.

**Table II:** Association between miR-339-5p relative expression and clinicopathological variables in lung cancer tissues.

		miR-339-5p expression (Ca/N)		
		
	n	Low expression	High expression	P-value
Lymph node metastasis				
Yes	23	21	2	
No	37	16	21	<0.001^a^
Gender				
Male	41	26	15	
Female	19	11	8	0.682^b^
Age				
≤60	41	25	16	
>60	19	12	7	0.872^b^
Pathological type				
Adenocarcinoma	39	24	15	0.978^b^
Squamous cell carcinoma	21	13	8	
Clinical stage				
I	25	6	19	<0.001^c^
II	11	9	2	
III	20	18	2	
IV	4	4	0	

a Two-sided Fisher’s exact test;

b χ^2^ test;

c Mann-Whitney test.

miR, microRNA; Ca, cancer; N, normal.
